# C-reactive Protein-to-Albumin Ratio: A Useful Predictor for Biliary Fistula After Hepatectomy

**DOI:** 10.7759/cureus.60735

**Published:** 2024-05-21

**Authors:** Takeshi Utsunomiya, Jota Watanabe, Kazunori Tokuda, Yoshitomo Ueno, Jun Hanaoka, Shigehiko Yagi, Fujimasa Tada, Atsushi Hiraoka, Tomoyuki Ninomiya, Hiromi Ohtani

**Affiliations:** 1 Department of Gastrointestinal Surgery, Ehime Prefectural Central Hospital, Matsuyama, JPN

**Keywords:** early postoperative complication, pobl, hepatectomy, hcc, car

## Abstract

Introduction

Postoperative bile leakage (POBL) has emerged as a complication following hepatectomy. POBL is associated with an elevated risk of liver failure and surgical death. This study aimed to examine risk factors for POBL in primary hepatocellular carcinoma (HCC) patients.

Methods

A total of 296 patients who had surgical resection for a preoperative diagnosis of primary HCC from January 2013 to December 2022 at Ehime Prefectural Central Hospital were included in this study. The patients were categorized into two groups based on the presence of POBL. The preoperative, operative, and histopathological findings were analyzed between the two groups. Risk factors were determined using multivariable analysis.

Results

Regarding preoperative findings, statistically significant differences were observed in white blood cell count, platelet count, C-reactive protein (CRP) level, and CRP-to-Albumin ratio (CAR) between the two groups (p = 0.023, p = 0.025, p = 0.011, and p = 0.012, respectively). As for intraoperative variables, only operation time (p = 0.017) was statistically correlated with the risk of POBL. Regarding pathological variables, there were no statistically significant differences between the two groups.

The optimal cut-off value of CAR, as determined by ROC curve analysis, was 0.053. This value had a sensitivity of 80.0% and a specificity of 72.8%.

Multivariate logistic regression analysis indicated that CAR ≥ 0.053 (p = 0.030) and operation time ≥ 308 min (p = 0.023) were independent potential markers for POBL after hepatectomy.

Conclusion

A high CAR level can be an effective predictor for POBL following hepatectomy.

## Introduction

Hepatectomy is one of the curative therapies for hepatocellular carcinoma (HCC) [1]. Liver resection is a procedure that requires advanced surgical skills, but the safety of the procedure has improved more than ever before due to better preoperative assessment of liver function, more standardized procedures, and advances in devices [2]. Due to advancements in perioperative management, the incidence of post-hepatectomy complications and mortality rates continue to decline. Postoperative bile leakage (POBL) is one of the complications after hepatectomy. The rate of POBL after hepatectomy has been reported to be 2.9%-11.0% [3-5]. POBL is well-recognized to be associated with a high risk of liver failure and surgical death [6]. As a result, identifying the risk factors for POBL and preventing them can enhance the quality of life of patients. Previous reports have indicated that direct bilirubin measurement in drainage fluid 3 days after hepatectomy is useful in predicting POBL [7]. Our institution does not routinely measure direct bilirubin in drainage fluid, and other factors that may be useful in predicting POBL were investigated in this study.

## Materials and methods

Patients

**Figure 1:**
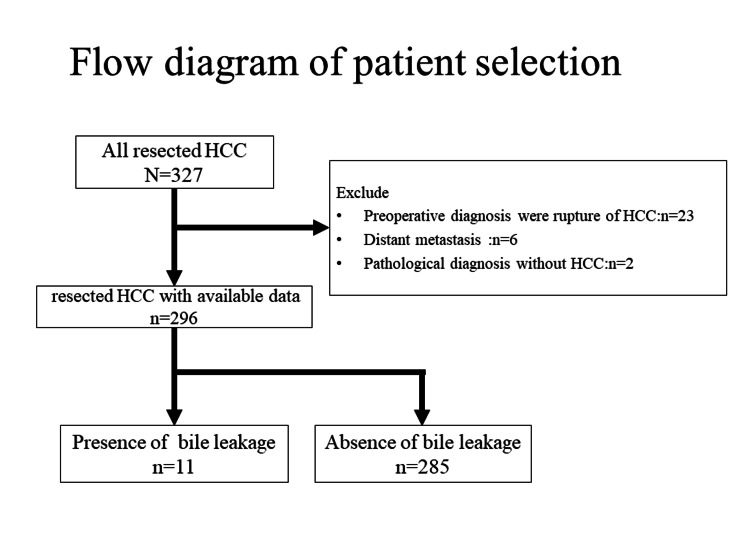
Flow diagram of patient selection.

A total of 327 patients with preoperatively diagnosed primary HCC who underwent surgical resection at Ehime Prefectural Central Hospital from January 2013 to December 2022 were enrolled in the study. The HCC diagnoses were routinely verified histopathologically from the resected specimens. Thirty-one patients were omitted from this research: 23 patients whose preoperative diagnosis was a rupture of HCC, six patients with distant metastasis, and two patients whose postoperative pathological diagnoses were not HCC. As a result, the study ultimately comprised 296 patients. The 296 patients were categorized into two groups based on the presence or absence of POBL. In this study, POBL was found in 11 of the 296 (3.7%) patients (Figure 1).

Data were sourced from prospectively maintained hospital databases. Surgical resection was recommended based on the Japanese Society of Hepatology HCC guidelines. Differences between the POBL and non-POBL groups were examined retrospectively with regard to preoperative data (clinical and laboratory data), surgical treatment data, and pathological findings.

POBL definitions

POBL was defined by the International Study Group of Liver Surgery (ISGLS) as patients with a drainage fluid bilirubin value greater than three times the serum bilirubin value on postoperative day (POD) 3 or later, or patients requiring intervention due to bile collections or biliary peritonitis [8]. According to the ISGLS definition, a grade A bile leakage was defined as a leakage not impacting the patient’s clinical management, a grade B bile leakage was defined as leakage necessitating active therapeutic intervention, and a grade C bile leakage was defined as a leakage requiring reoperation. In this study, the numbers of cases with grade B or grade C bile leakages were counted.

Surgical procedures

The Pringle maneuver involved a 20-minute compression of the hepatoduodenal mesentery and a 5-minute release.

An ultrasonic cavitation device (CUSA; Valleylab, Boulder, CO, USA) was regularly employed for liver parenchymal transection during open and laparoscopic procedures. The CUSA was primarily used for these procedures. The LigaSure™ vessel sealing system and clips were also used for laparoscopic surgery.

All patients underwent drain placement at the hepatectomy site.

Protocol

Collection of Clinical and Laboratory Data

Clinical data were collected concerning patient demographics (gender and age), anthropometric parameters (height, weight, and BMI), as well as laboratory data such as preoperative HbA1c, CRP, and albumin values as preoperative parameters.

CAR Definition

CAR was estimated using preoperative CRP and serum albumin values. These data were obtained preoperatively and calculated using the following formula: CAR = CRP (mg/dL) / Serum albumin (g/dL) [9].

Collection of Surgical Treatment Data

Operation time, hepatectomy type, blood loss, availability of blood transfusions, and resected liver weight were analyzed.

Regarding the type of hepatectomy, the study also took into account whether the hepatectomy was central. Central hepatectomies were defined as follows: mesohepatectomy (segments (Sgs) 1, 4, 5, and 8), right anterior sectionectomy (Sgs 5 and 8), and other operations that were surgically similar to these procedures [10, 11].

Collection of Pathological Findings

Based on the General Rules for the Clinical and Pathological Study of Primary Liver Cancer (March 2019) [12], the pathological findings (tumor size, number of HCC, presence of vascular invasion, etc.) were analyzed. Regarding tumor diameter, the largest diameter was utilized if there were multiple tumors.

Statistical analysis

Continuous variables are expressed as medians with ranges and were compared using Mann-Whitney U tests. The cut-off values for predicting POBL were determined through receiver operating characteristic (ROC) curve analysis. Significant variables in univariate analyses that were associated with POBL on Mann-Whitney U tests, Pearson’s χ2 test, or Fisher’s exact tests were included in univariate and multivariate analyses using step-down logistic regression and likelihood tests. Regression models were calibrated using Hosmer-Lemeshow tests. Significance was defined as p < 0.05. All data were examined utilizing SPSS® version 25 (IBM Corp., Armonk, NY, USA).

Ethics statements

The institutional review board of Ehime Prefecture Central Hospital approved this study (Approval number: 31-65), which was conducted in accordance with the ethical standards established in the Declaration of Helsinki in 1995 (Brazil 2013 revision). Written, informed consent was obtained using the opt-out principle. The nature of the study and the right to refuse participation were disclosed to the public online. None of the authors has any conflicts of interest to disclose regarding this study.

## Results

POBL was grade B in 10 cases and grade C in one case. The demographic and baseline characteristics of patients with and without POBL were compared (Table 1). No statistically significant differences were observed in age, gender, BMI, smoking history, Child-Pugh classification, ALBI score, tumor marker, cirrhosis, etiology of cirrhosis, presence of comorbidities, and presence of diabetes mellitus, excluding white blood cell count, platelet count, CRP level, and CAR, between the two groups (p = 0.023, p = 0.025, p = 0.011, and p = 0.012, respectively).

**Table 1:** Preoperative data in patients with or without POBL. Continuous variables are presented as the medians with ranges. Categorical variables are presented as the patient numbers and ratios (%). Hb: Hemoglobin; Plt: Platelet;  NLR: Neutrophil-to-lymphocyte ratio; PNI: Prognostic nutritional index; PT: Prothrombin time; AST: Aspartate aminotransferase; ALT: Alanine aminotransferase; T-Bil: Total bilirubin; Alb: Albumin; GNRI: Geriatric Nutritional Risk Index; CRP: C-reactive protein; CAR: C-reactive protein-to-Albumin ratio; AFP: Alpha-fetoprotein; DCP: Des-gamma-carboxy prothrombin; SUVmax: Maximum standardized uptake value; ALBI score: Albumin-bilirubin score; POBL: Postoperative bile leakage.

	POBL - N = 285	POBL + N = 11	P-value
Gender (Male)	226 (79.3%)	10 (90.9%)	0.309
Age	72 (38-93)	72 (59-80)	0.778
BMI	23.3 (13.9-42.2)	21.3 (19.1-27.4)	0.365
Smoke history	196 (68.8%)	9 (81.8%)	0.289
WBC	5190 (1730-13760)	5910 (3990-15370)	0.023
Hb	13.7 (6.1-17.7)	14.5 (10.6-16.6)	0.974
Plt	15.4 (4.0-42.6)	19.5 (11.3-38.2)	0.025
NLR	2.1 (0.6-8.6) N=252	2.9 (1.4-5.1) N=10	0.190
PNI	47.2 (30.0-63.3) N=252	48.5 (41.3-57.1) N=10	0.601
PT	92.0 (31.0-132.0)	90.0 (61.0-109.0)	0.435
AST	36 (11-243)	27 (15-60)	0.314
ALT	29 (4-312)	17 (10-121)	0.055
T-Bil	0.7 (0.2-2.9)	0.7 (0.3-2.1)	0.428
Alb	4.0 (2.5-5.1)	4.1 (3.2-4.5)	0.993
GNRI	104.5 (72.2-135.2)	100.6 (92.5-113.5)	0.416
CRP	0.1 (0.01-13.38) N=261	0.79 (0.02-5.00) N=10	0.011
CAR	0.024 (0.0021-4.68) N=261	0.24 (0.0045-1.39) N=10	0.012
HbA1c	5.9 (4.0-11.8) N=283	6.4 (4.7-7.8) N=10	0.755
AFP	8.7 (1.0-772805.2) N=282	11.1 (1.4-132550.0)	0.495
DCP	91 (6-151330) N=282	384 (27-45782)	0.118
Tumor SUVmax	3.7 (2.0-24.3) N=182	8.5 (3.0-15.7) N=5	0.061
ALBI score	-2.68 (-3.67- -1.35)	-2.77 (-3.08- -1.95)	0.785
Child Pugh classification A / B / C	270 (94.7%) / 15 (5.3%) / 0	11 (100%) / 0 / 0	0.559
Cirrhosis	136 (47.7%)	3 (27.3%)	0.182
Etiology of cirrhosis virus / Non virus	90 (66.2%) / 46 (33.8%)	3 (100%) / 0	0.296
Comorbidities / Diabetes mellitus	246 (86.3%) / 113 (39.6%)	10 (90.9%) / 4 (36.4%)	0.548 / 0.546

Among intraoperative variables, only operation time (p = 0.017) was statistically associated with the risk of POBL (Table 2).

**Table 2:** Intraoperative variables in patients with or without POBL. Continuous variables are presented as the medians with ranges. Categorical variables are presented as the patient numbers and ratios (%). Hr3, Hr2, Hr1, HrS and Hr0 represented trisectionectomy, hemihepatectomy, segmentectomy, subsegmentectomy and limited resection, respectively. POBL: Postoperative bile leakage.

	POBL - N = 285	POBL + N = 11	P-value
Operative time (min)	252 (101-790)	331 (203-527)	0.017
Blood loss (ml)	377 (0-17930)	480 (20-3140)	0.473
Resected liver weight (g)	132 (1-1748)	224 (61-957)	0.083
Transfusion	64 (22.5%)	5 (45.5%)	0.085
Laparoscopic operation	76 (26.7%)	2 (18.2%)	0.405
Hr3	1 (0.4%)	0 (0%)	0.963
Hr2	37 (13.0%)	3 (27.3%)	0.174
Hr1	64 (22.5%)	3 (27.3%)	0.471
HrS	77 (27.0%)	3 (27.3%)	0.609
Hr0	106 (37.2%)	2 (18.2%)	0.168
Central hepatectomy	83 (29.1%)	5 (45.5%)	0.201

Regarding pathological variables, there were no statistically significant differences between patients with POBL and those without POBL (Table 3).

**Table 3:** Pathological variables in patients with or without POBL. Continuous variables are presented as the medians with ranges. Categorical variables are presented as the patient numbers and ratios (%). Greater than 0 (>0) means that infiltration is positive. Fc: Formation of capsule; Fc-inf: Infiltration of capsule; Sf: Septal formation; S: Serosal infiltration; Vp: Portal vein infiltration more including branches; Vv: Hepatic vein infiltration including branches; Va: Hepatic artery infiltration including branches; B: Bile duct infiltration including branches; Im: Intrahepatic metastasis; SM: Infiltration of surgical margin; POBL: Postoperative bile leakage.

	POBL - N = 285	POBL + N = 11	P-value
Tumor size	33 (5-220)	30 (1-170)	0.736
Fc>0	168 (58.9%)	6 (54.5%)	0.496
Fc-inf>0	116 (40.7%)	6 (54.5%)	0.28
Sf>0	153 (53.7%)	5 (45.5%)	0.574
S>0	29 (10.2%)	2 (18.2%)	0.323
Vp>0	55 (19.3%)	4 (36.4%)	0.159
Vv>0	28 (9.8%)	2 (18.2%)	0.311
Va>0	0	0	-
B>0	6 (2.1%)	1 (9.1%)	0.236
Im>0	40 (14.0%)	1 (9.1%)	0.534
Sm>0	13 (4.6%)	1 (9.1%)	0.422

**Figure 2:**
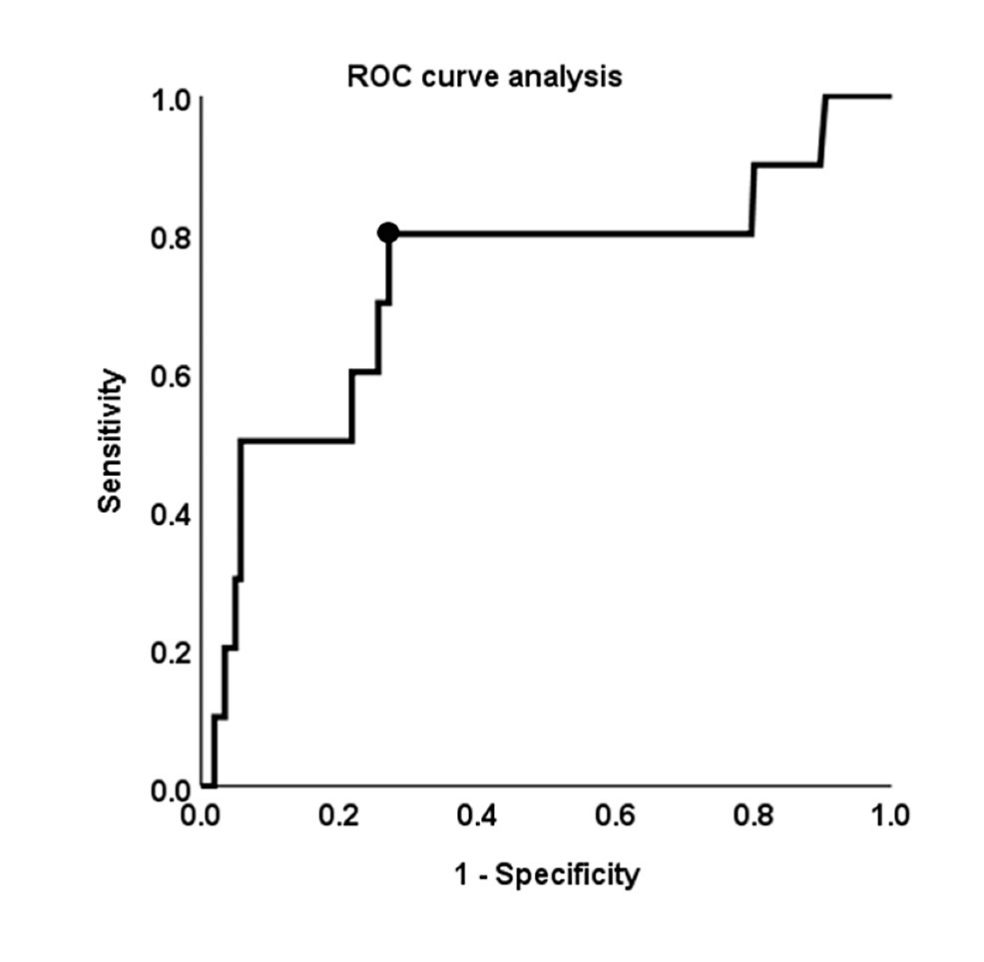
Receiver operating characteristic (ROC) curve analysis of the CAR for predicting POBL. The area under the ROC curve of CAR is 0.734. The optimal cut-off value of CAR is determined to be 0.053. The sensitivity and specificity are 80.0% and 72.8%, respectively. POBL: Postoperative bile leakage.

The optimal CAR cut-off value was determined by ROC curve analysis. The most appropriate cut-off value for assessing the risk of POBL was identified through ROC curve analysis (Figure 2).

With an area under the curve of 0.734, the most appropriate cut-off value was 0.053. This value showed a sensitivity of 80.0% and a specificity of 72.8%. The incidence rate of POBL was significantly higher in patients with higher CAR (CAR ≥ 0.053, N = 79) at 10.1% (8/79) than in those with lower CAR (CAR < 0.053, N = 192) at 1.0% (2/192) (p = 0.001). Similarly, other cut-off values that were extracted by univariate analysis were computed using ROC curve analyses.

Multivariate logistic regression analysis revealed that CAR ≥ 0.053 (p = 0.030) and operation time ≥ 308 min (p = 0.023) were independent potential markers for POBL after hepatectomy, as depicted in Table 4.

**Table 4:** Univariate and multivariate logistic regression analyses of predictors of POBL. CRP, C-reactive protein; CAR, C-reactive protein-to-Albumin ratio; Plt, platelet; POBL: Postoperative bile leakage.

Parameters	Univariate		Multivariate	
	Odds ratio (95% CI)	P-value	Odds ratio (95% CI)	P-value
WBC ≥ 5705 (/µl)	4.242 (1.102-16.335)	0.036	–	–
Plt ≥ 16.8 (10^4^/µl)	4.180 (1.086-16.094)	0.038	–	–
CRP ≥ 0.22 (mg/dl)	10.301 (2.137-49.658)	0.004	–	–
CAR ≥ 0.053	10.704 (2.220-51.621)	0.003	6.133 (1.194-31.499)	0.030
Operative time ≥ 308 (min)	6.167 (1.749-21.739)	0.005	5.320 (1.260-22.456)	0.023

## Discussion

The reported rate of POBL categorized as ISGLS grade B and C after hepatectomy typically ranges from 2.9%-6.5% [3, 4]. This makes the POBL rate of 3.7% in this study a reasonable figure.

There are various reports about the risk factors of POBL. For example, BMI > 28 kg/m², history of hepatectomy, biliary reconstruction, male sex, diabetes, left trisectionectomy, central hepatectomy, extended hemihepatectomy, segment I hepatectomy, intraoperative blood transfusion, anatomical hepatectomy, intraoperative bleeding ≥1,000 ml, and long operative time were identified as risk factors of POBL [13-17].

This study showed preoperative CAR ≥0.053 and operative time ≥308 min were independent risk factors for POBL. Previous reports have revealed that long operative time was an independent risk factor for POBL [13-15]. However, there are no reports about the correlation between CAR and POBL. As a preoperative nutritional indicator, PNI has also been reported in the past as a factor associated with POBL [18]. In that study, about 50% of non-HCC liver resection cases were included, and the background of the target patients differed significantly from the present study, which included only primary HCC cases. In this study, PNI as a preoperative nutritional indicator was not considerably different as a factor for POBL, and only CAR was identified as an independent factor.

CAR consists of the CRP level to serum albumin level ratio [4].

CRP is one of the most well-known markers of inflammation. Serum CRP concentrations are determined by the synthetic rate of its production in the liver, which is predominantly regulated by interleukin-6 [19]. Preoperative low serum Albumin level was reported as a risk factor for intra-abdominal infection after major hepatectomy [20]. Additionally, inflammation is widely recognized to prolong wound healing processes.

An increase in CRP level with a decrease in Albumin level has been found in many cancers [21]. Serum CRP and albumin are two crucial acute-phase proteins that reflect the nutritional status during the systemic inflammatory response, which commonly occurs in malignant tumors [22]. So, it is easy to imagine that dividing CRP level by Albumin level could be a better indicator of inflammation and nutritional status than CRP level alone or Albumin level alone. This may enable preoperative detection of high-risk groups with POBL relevant to patient prognosis and may result in enhanced preoperative nutritional conditions and inflammatory findings, more careful surgical approaches, effective drain placement, and C-tube placement when necessary, leading to enhanced patient outcomes.

Some studies indicated that CAR was a valuable prognostic factor for HCC. Higher CAR levels (≥ 0.037) were reported to be related to poor overall survival [23, 24]. Other reports indicated that the optimal cut-off value of CAR was 0.11, and the elevated CAR group had a significantly poor prognosis for OS and DFS [25]. Regarding HCC patients undergoing transarterial chemoembolization, a higher CAR level (≥ 0.06) was reported to be correlated with overall survival [26]. The cut-off values for CAR varied between reports, ranging from 0.037 to 0.11.

The current study also analyzed the CAR ≥ 0.053 and CAR < 0.053 groups concerning overall survival and recurrence-free survival, but no substantial differences were observed.

This dissociation in cut-off values could be attributed to various factors, such as differences in the background of the targeted patients and treatment options available in case of recurrence during follow-up. Considering this, the present analysis concentrates on the postoperative complication of POBL, and the cut-off values for CAR may be less variable compared to those associated with OS and DFS.

This study has several limitations. First, the inclusion of only 11 cases of biliary fistula is noted. It is known from previous reports that the incidence of biliary fistula is reducing, and a larger number of cases would enable a more precise analysis.

Additional limitations include the fact that the study was limited to initial occurrences of HCC, excluding cases of rehepatectomy, which can be a risk factor for POBL, and that it was a retrospective study of consecutive cases at a single center only. To address these limitations, further validation of the findings of this study in rehepatectomized cases and collaborative studies at other centers is required.

## Conclusions

The present study demonstrated that a high CAR level can be an effective predictor for POBL following hepatectomy. CAR is simple and easy to calculate from CRP and Albumin, which are usually included in the biochemical parameters performed in the preoperative examination. The greatest advantage demonstrated in this study is that a simple preoperative blood examination can easily identify the high-risk group for POBL. In the group of patients with high CAR (≥ 0.053), POBL may be reduced by enhancing the preoperative nutritional status and optimizing surgical planning to minimize operation duration. Since this is a retrospective study, further prospective studies are needed to validate the usefulness of the CAR cutoff values obtained in this study.
